# Using participatory action research to pilot a model of service user and caregiver involvement in mental health system strengthening in Ethiopian primary healthcare: a case study

**DOI:** 10.1186/s13033-022-00545-8

**Published:** 2022-07-11

**Authors:** Sisay Abayneh, Heidi Lempp, Brandon A. Kohrt, Atalay Alem, Charlotte Hanlon

**Affiliations:** 1 College of Health Sciences, School of Medicine, Department of Psychiatry, Addis Ababa University, WHO Collaborating Centre in Mental Health Research and Capacity Building, Addis Ababa, Ethiopia; 2 Madda Walabu University College of Education and Behavoural Studies, Bale Robe, Ethiopia; 3 Centre for Rheumatic Diseases, School of Immunology and Microbial Sciences, Faculty of Life Sciences and Medicine, Weston Education Centre, King’s College London, 10, Cutcombe Rd, London, SE5 9RJ UK; 4 Department of Psychiatry, George Washington University, Washington, DC USA; 5 Centre for Global Mental Health, Institute of Psychiatry, Psychology and Neuroscience, King’s College London, 16 De Crespigny Park, London, SE5 8AF UK; 6 Centre for Innovative Drug Development and Therapeutic Trials for Africa (CDT-Africa), College of Health Sciences, Addis Ababa University, Addis Ababa, Ethiopia

**Keywords:** Theory of change, Participatory action research, Sub-Saharan Africa, Service-user involvement

## Abstract

**Background:**

Little is known about actual involvement or how to achieve service user and caregiver in mental health systems strengthening in low-and middle-income countries. This study describes the processes and explores involvement experiences of participants in a pilot study of a new model of service user involvement in mental health system strengthening in a rural district in southern Ethiopia.

**Methods:**

We applied a case study design using participatory action research (PAR). The PAR process comprised of three stages, each with iterative activities of plan, act, observe and reflect. Two stakeholder groups, a Research Advisory Group (RAG) and Research Participant Group (RPG), were established and collaborated in the PAR process. Data collection involved process documentation of meetings and activities: attendances, workshop minutes, discussion outputs, reflective notes, participatory observation of sessions, and in-depth interviews with 12 RPG members. We analyzed the process data descriptively. Thematic analysis was used for qualitative data. Triangulation and synthesis of findings was carried out to develop the case study.

**Results:**

The stakeholder groups identified their top research priorities, developed an intervention and action plan and made a public presentation of preliminary findings. Key mechanisms used for inclusive participation included capacity building and bringing together diverse stakeholders, anchoring the study in established strong community involvement structures, and making use of participatory strategies and activities during the PAR process. Four themes were developed about experiences of involvement in PAR: (i) expectations and motivation, (ii) experiences of the dynamics of the PAR process, (iii) perceived impacts of involvement in the PAR process, and (iv) implementation challenges and future directions.

**Conclusions:**

This case study demonstrated the feasibility and acceptability of implementing a complex model of service-user involvement in mental health system strengthening in a resource constrained setting. More needs to be done to embed service-user involvement into routines of the primary healthcare system, alongside sustained support and strengthening multi-stakeholder collaboration at multiple levels.

**Supplementary Information:**

The online version contains supplementary material available at 10.1186/s13033-022-00545-8.

## Background

Mainstreaming service user and caregiver involvement (hereafter service-user involvement) is increasingly acknowledged as a promising strategy to scale-up quality mental health services in low-and-middle-income countries (LMICs) [1, 2]. Nonetheless, little is published about how to actually involve service-users and their experiences of involvement in LMICs [1–3]. Multiple factors hinder the uptake of service-user involvement within mental health systems in LMICs, including low policy priority, poor infrastructure and funding, stigma and discrimination, and human rights abuses [1, 4]. Mental health care systems are not modeled on service-user driven approaches [5]; there is a lack of strategies/models that could guide how best to implement service-user involvement[1–3], and little attention has been given to the empowerment and mobilization of service-users [1, 6, 7].

In the Ethiopian mental health system, service-user involvement is a new concept, and is influenced by complex intersecting factors within and beyond the healthcare system. There is limited experience and awareness of how to address that complexity and achieve a workable model of involvement [8]. To fill this gap, a generic Theory of Change (ToC) model for service-user involvement in mental health system strengthening has been developed [9].The model depicts service-user involvement in mental health system strengthening in a general way and leaves the potential focus areas (e.g., advocacy, service delivery, research, education) to be specified. The ToC also lacks an appropriate methodological approach to foster active and inclusive involvement of all stakeholders, especially to overcome power dynamics that typically exclude service-users [10, 11].

In order to address these gaps and provide a more inclusive participatory and contextually useful ToC, we choose to use a Participatory Action Research (PAR) approach as a heuristic to support implementation of the ToC as detailed elsewhere [12]. This is in line with theory of action, which advocates for the need to insert action mechanisms into programs to activate ToCs[13]. The iterative cyclical activities of PAR (plan, act, observe, and reflect) are promising strategies to facilitate learning about what, how and why change is unfolding [13]. PAR offers possibilities for equitable active participation of diverse stakeholders, and has been recommended as a suitable approach to explore, understand, and provide solutions to emerging, complex, contextual issues, including system needs in public health [14, 15].

In this study, we describe the PAR process and explore participants’ experiences of involvement in PAR as a case study to evaluate the pilot of a model of service-user involvement in mental health system strengthening in rural primary healthcare in southern Ethiopia.

## Methods

### Setting

This study formed part of a larger research project aiming to develop service-user involvement in mental health system strengthening in Ethiopia. This was initiated as part of the ‘Emerging mental health systems in low-and-middle-income countries’ (Emerald) project including six LMICs(Ethiopia, India, Nepal, Nigeria, South Africa and Uganda) [16]. Previously, we conducted formative qualitative exploration of the experiences, perceived barriers and facilitators to service-user involvement in mental health system strengthening [8] and a ToC was developed [9].The current study is part of the pilot implementation of the model.

This work was conducted in Sodo district of the Gurage Zone of the Southern Nations, Nationalities and Peoples’ region, southern Ethiopia. The capital town of the district, Buie, is located about 100 km from Addis Ababa. At the time of the study, the district had 58 *Kebeles* (sub-districts); four urban and 54 rural. The population of the district was about 170,000. Health care was provided by one primary hospital, eight primary health centers and 58 health posts. This pilot was linked to the primary hospital, situated in Buie town. Service-users from eight primary healthcare clinics had been referred to the psychiatric nurse-led clinic at Buie for mental health care. The hospital was selected for pilot implementation together with local stakeholders considering accessibility (walking distance) and the higher service-user caseloads compared to primary health centers.

### Design

The pilot implementation process was guided by a PAR approach situated in critical social theory; details of the theoretical background have been described in the study protocol [12], briefly described here. PAR was selected as a suitable approach, previously applied to this area and providing practical strategies for involvement of service-users in numerous domains of mental health systems (e.g., planning, research, service quality improvement, and advocacy) [12]. Critical social theory-informed PAR, which positions PAR within a critical onto-epistemology (Critical Participatory Action Research; hereafter, CPAR), provides a theory-informed basis for the kinds of relationship that need to be developed among individuals, institutions and other key stakeholders in a particular initiative [12]. CPAR acknowledges the marginalization of service-users and seeks to create a communicative space for ethical relationships. An approach such as CPAR is required to examine, expose and alter the unacknowledged social inequalities, structural and power injustices that are experienced by service-users towards empowerment and partnership [12].

We used a qualitative case study approach to describe the process and to explore the experiences of participants in an ongoing pilot of service-user involvement in mental health system strengthening in rural Ethiopian primary healthcare (the unit of analysis for this study) [17, 18]. Case studies can directly inform assessments of where, when, how and for whom interventions might be successfully implemented, and to consolidate learning on how interdependencies, emergence and unpredictability can be managed to achieve and sustain desired effects [17, 18]. Moreover, a case study offers a flexible approach (by incorporating different paradigmatic positions and use of multiple data sources and methods) that enables holistic, in-depth, multiple perspectives to examine and understand a complex phenomenon within a natural setting from the perspective of those involved [17]. A case study can offer considerable potential for strengthening faith in both external and internal validity [18]. The study was undertaken between March 2018 and January 2020.

### Participants

 At the beginning of the PAR process, we established two stakeholder groups: a Research Advisory Group (RAG) and a Research Participant Group (RPG). Recruitment of members was using a purposive maximum-variation sampling strategy to ensure adequate representation from a broad range of stakeholders. Most RAG members were recruited from an existing project Community Advisory Board (CAB) [19]. The RAG was comprised of various stakeholders who contributed to co-production of the ToC and were involved in advising on the priority problems for improving mental health and supported the initiative to strengthen RPG involvement in mental health. The RPG consisted of service users, caregivers and health professionals. They participated throughout the research processes. The RAG and RPG met twice during the course of the study (two full days during Stage 1 and one full day in Stage 3).

Table 1 presents a summary of the composition of the stakeholder groups; more details about inclusion criteria and roles of the stakeholder groups have been described previously [12].

**Table 1:** Composition of stakeholder groups

Composition of research advisory group	No	Composition of research participant group	No
Health sector	5	Service users	6
Justice sector	3	Caregivers	4
District administration	2	Health professionals	2
Education	1		
Labor and social affairs	1		
Gender, youth and children affairs	1		
Community organizations (Religion, Idir)	2		
Volunteer activists	2		
Service users	5		
Caregivers	4		

### Data collection

We gathered data through various sources and methods, including process documentation, participatory observations and in-depth interviews (See Table 2).

**Table 2:** Summary of data collection methods

Methods of data collection	Description
Process documentation	• Workshop (n = 3) participant attendances and PAR sessions(n = 11) • Workshop minutes(n = 3) and audio-recordings(n = 11) • Summary of prioritization exercise outputs, review of flipchart notes, and photographs • Facilitator reflection notes
Participatory observation	• Participatory observation of workshops (n = 3) and PAR sessions (n = 11). Data collection focused on: interaction between participants/group dynamics, role of participants, agenda items discussed
In-depth interviews	• Conducted face-to-face interview with 12 members of RPG to explore experiences of involvement in the PAR process using a topic guide (Additional file 1) • Participants were fully informed about the study and gave informed written consent (finger print if non-literate) • All interviews were conducted by a single interviewer (female) with a master’s degree, who had not been engaged in the research process • All interviews were audio-recorded • Interviews lasted between 23 and 49 min (with an average of 32 min)

### Data analysis

The data analysis was descriptive for the processes of the PAR, and thematic analysis for RPG experiences of involvement (See Table 3). The PAR descriptive data analysis process continued throughout the study as an iterative process. PAR offers several methods and techniques (e.g., charts and diagrams), which function as a powerful mechanism to facilitate active engagement of participants (e.g., in the case of literacy and/or numeracy challenges) in data generation, analysis and synthesis [20].

**Table 3:** Summary of data analysis methods

PAR process description	Experiences of involvement
Data analysis was participatory that engaged participants: • Members of RPG and RAG identified, categorized and summarized top priorities using nominal group techniques [12] • Members of RPG generated long lists of data in small groups in a two-times per weekly PAR sessions using Venn diagrams and flipcharts • SA conducted preliminary thematic analysis of from meeting minutes, flipchart notes and facilitators reflective notes • Then shared with RPG by displaying on the wall to demystify the PAR process, enable collective sense-making of the data, encourage their active participation, ensure an accurate representation of their views and critically reflect on any gaps • Finally, the summary of the prioritization exercise and flipchart presentations at each session was triangulated with audio-recordings of sessions, minutes of the workshops and the SA's reflective notes; and presented descriptively for the three stages of PAR (See Figs. 3, 4)	RPG experience of involvement data was analyzed using inductive and deductive approach using six steps thematic analysis procedures described by Braun and Clarke [21]: • All interviews were transcribed verbatim into Amharic by independent transcribers • SA checked transcripts for accuracy, and translated into English • De-identified transcripts were then uploaded to Opencode 4.03 software, to assist data management and analysis • SA and a colleague independently carried out initial coding of two randomly selected transcripts inductively • Following discussion and consensus about the coding, SA coded all the transcripts and collated the codes into sub-themes and themes • The coding trees developed from the in-depth interviews data were used to link the content and common elements from supporting datasets (field notes, workshop minutes and reflective notes, observation) through a deductive thematic analysis approach [21] using a process of describe-compare-relate [22]

**Fig. 1:**
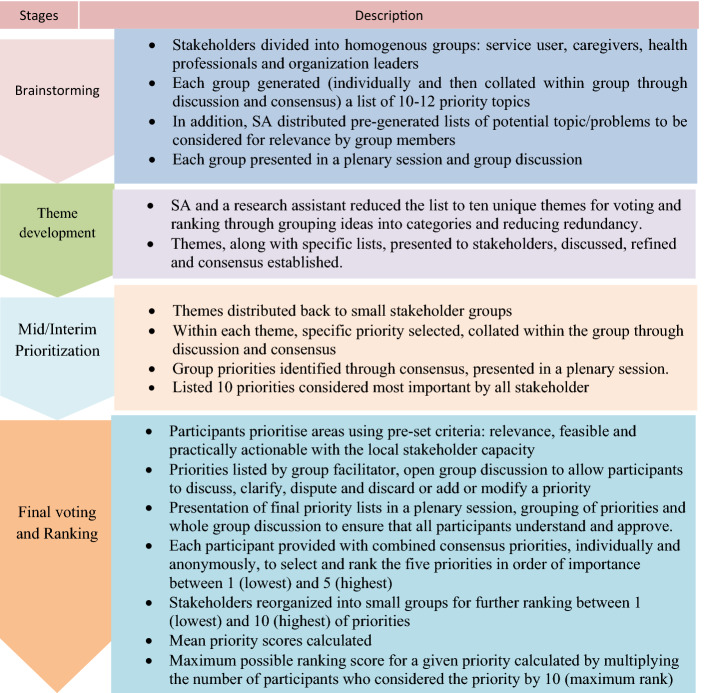
Summary of stage 3 PAR processes

**Fig. 2:**
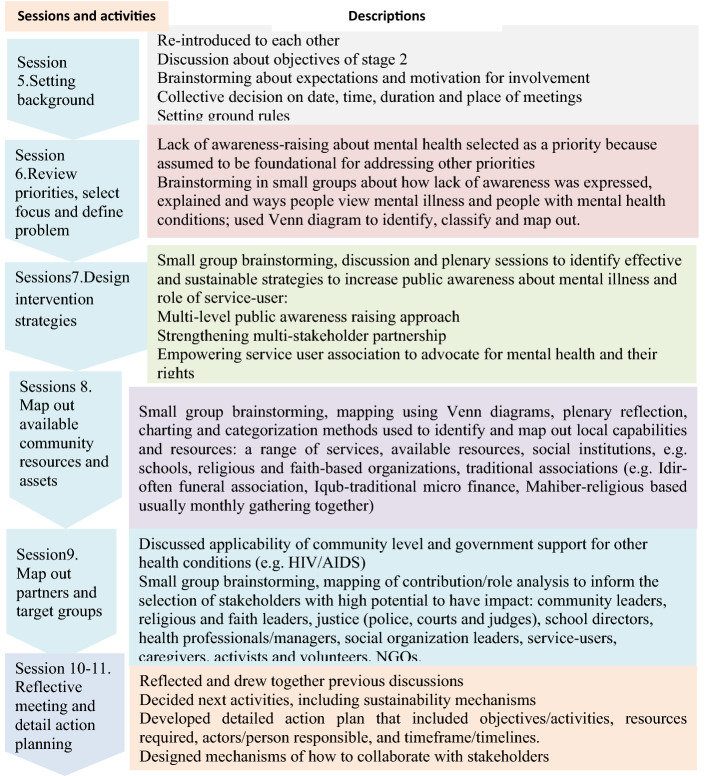
Steps in prioritization exercise

**Fig. 3:**
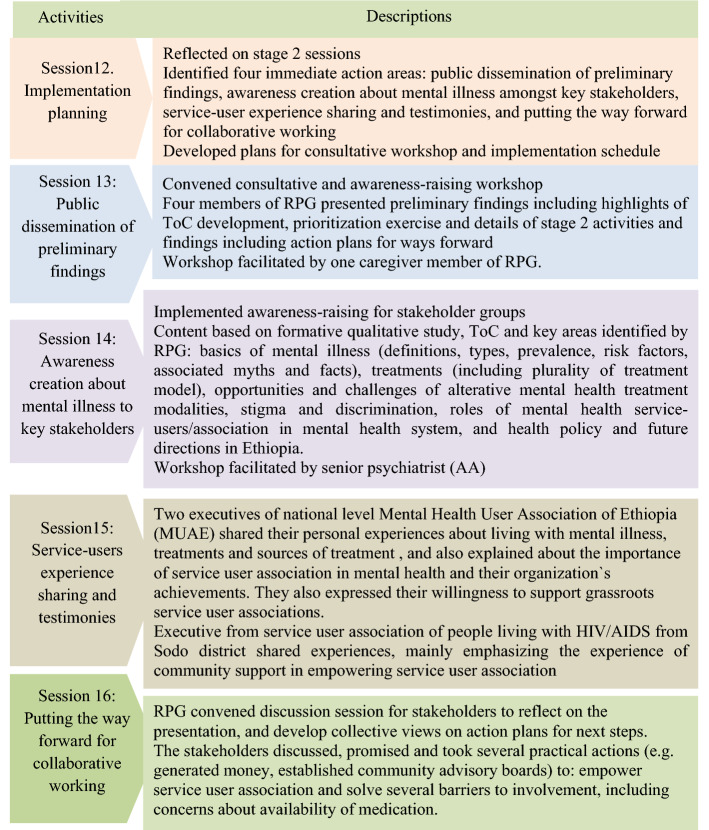
Summary of Stage 2 activities

**Fig. 4:**
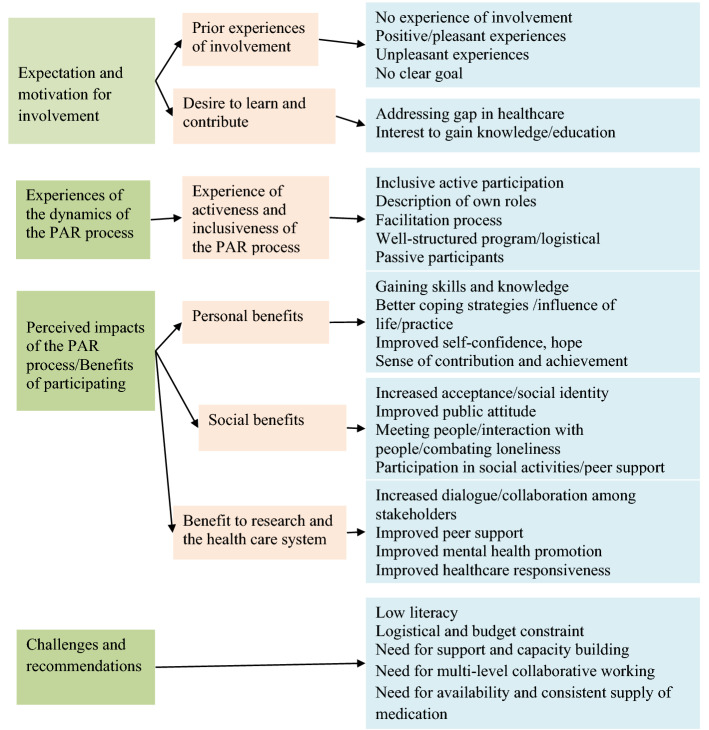
A thematic map

### Rigour

To enhance trustworthiness of data, we used several strategies. Many scholars have reported challenges in ensuring rigour in PAR because of the diversity of approaches and types of PAR. PAR cannot ignore the traditional validity requirements (e.g., validity, reliability, trustworthiness), but these alone are inadequate to judge the quality of PAR because of its distinct ontological and epistemological stance [23]. Within the cyclical collaborative decision-making process in PAR, the participants drive processes that make the content of interventions unpredictable. Hence, the mainstream notions of fidelity need to be reframed to respond to the complexity, culturally situated, and constantly changing (emergent nature rather than predetermined intervention protocol) circumstances of PAR interventions [24]. According to Trickett et al. three main factors pose challenges in using traditional fidelity conceptualizations in PAR: (i) the goal is not only scientific but also social action on local issues, (ii) engagement of various stakeholders/partners by itself is part of the intervention, which affect both processes and outcomes, and (iii) goals include community level as well as individual level changes [25]. In this study, we utilized quality criteria adopted from several studies which consisted of elements of qualitative study, case study and PAR approaches [23, 26].These quality criteria included: credibility, transferability, dependability, conformability, consistency/construct validity, outcome validity, process validity, democratic validity, dialogical validity, and catalytic validity (See Additional file 1 for details).

### Reflexivity and positionality

 The co-authors have previous experiences of participatory research, are mental health researchers, and have multi-disciplinary backgrounds (psychiatry, psychology, sociology, and epidemiology), which facilitated critical reflection and contextualized interpretation of the data. AA facilitated the workshops and SA facilitated the field PAR activities.

Being aware of the obvious differences in position between participants and SA (coming from university, educated, and experience in research); SA made a deliberate attempt to equalize power with participants through making participants aware that each of them had lived experiences and roles to play in knowledge production. SA used non-technical language so that all participants would understand the PAR process; encouraged participants to share experiences and express opinions non-judgmentally (positive or negative) and gave persistent affirmation to participants’ ideas. As sessions progressed, participants were encouraged to take on more active roles, including leading the sessions, presenting findings, and facilitating workshops. SA acted as a mediator to create mutual understanding and agreement where consensus was absent and to create opportunities for critical dialogue among participants to enhance their understanding through questioning and help participants to challenge long-held myths about mental illness that tend to be sustained [27].

## Results

The results are presented in two sections. In the first section, we describe the key activities involved in the three stages of PAR to pilot the model. In the second section, we present the analysis of the experiences of participants involved in the PAR process.

### Description of the PAR process

The PAR process case description covers activities in the three stages of PAR, each with cyclical activities of plan, act, observe and reflect (See Additional file 2): (i) Establishing partnership, capacity building, and prioritization exercise, (ii) program development and action planning, and (iii) Implementation and process evaluation.

### Stage 1: establishing a partnership, capacity building and prioritization exercise (March 2018–August 2019)

During this first stage, we established the RPG and RAG. In our ToC, building capacity of stakeholders was a key intervention to facilitate involvement [9]. Accordingly, prior to involvement and during the PAR process, the stakeholder groups were equipped and empowered through participatory training and consultative workshops. The trained service users and caregivers established a service user association with 24 members (12 service users and 12 caregivers), the first grassroots association in Ethiopia. We also convened three consultative workshops and capacity building sessions to create a receptive community environment, involving engagement with diverse stakeholder groups in addition to the RPG and RAG (n = 47); See Table 4 for stakeholder characteristics. The objectives of the training courses, consultative workshops and activities are briefly summarized in Additional file 3.The stakeholders collaborated with the researchers throughout the PAR process and actively contributed to the success of the study as insiders, being members of the community (See Figs. 1, 2, 3).

**Table 4:** Characteristics of stakeholders in the study

Types of Participants		Characteristics					
		Number of participants		Gender		Age range	Highest formal education
	Workshop 1	Workshop 2	Workshop 3	Male	Female	18–29, 30–49, 50+	None, Primary, Secondary, Tertiary
Government sector office leaders	8	16	18	13	5	18–30(3), 30–49(13), 50+(2)	BSC/BA(11), Diploma(6),MSC(1)
Community institution leaders(Idir leaders, Religious and faith-based)	6	–	3	7	–	50+(7)	Non-formal (4), BA (2), Diploma (1)
Health professionals	5	6	6	3	3	18–29(3),30–49(3)	Diploma(1), BSc(5)
Service-users	14	9	11	5	9	18–29(5), 30–49(5), 50+(4)	No literacy(4),None(3), primary(7)
Caregivers	14	6	8	7	7	18–29(3),30–49(9), 50+(2)	No literacy(7) None(1), primary(5), secondary(1)
Total	47	37	46	35			

To identify which specific aspect of mental health system strengthening was a priority for action, we convened a two day prioritization exercise with the stakeholders (n = 37) using principles of PAR based on Nominal Group Techniques, as detailed elsewhere [12] and summarized in Fig. 2. Participants identified their top ten concerns, which included multilevel lack of awareness about mental illness, and stigma and discrimination at the top of list (See Additional file 4).

### STAGE 2. Programme development and action planning, September–December 2019

The purpose of this stage was to develop interventions and action plans based on the priorities identified in stage 1. Members of the RPG, SA and a research assistant worked together over eleven biweekly sessions, each approximately two hours long, to explore the priorities in more depth, selected a priority, develop an intervention and action plan (See Fig. 2).

### Stage 3. Implementation and process evaluation, December 2019-January 2020

In stage3, the RPG conducted reflective and implementation strategy development sessions on how to apply the plan into doable action and reconvened a one-day consultative workshop (December 2019) with a broad range of stakeholders (See Table 4, workshop 3). An overview of stage 3 PAR processes is summarized in Fig. 3.

### Experiences of RPG involvement in PAR processes

In this section, we present the findings of the case study about the experiences of members of RPG involved in the pilot model. All service-user participants had a confirmed diagnosis of psychosis/bipolar, epilepsy, or alcohol use disorder (See Table 5 for demographic characteristics).The thematic analysis resulted in four main themes, illustrated with sub-themes and key codes in Fig. 4 and also see Additional file 5 for more illustrative quotes.

**Table 5:** Demographic characteristic of RPG members

Characteristics	Frequency
Gender	
Male	6
Female	6
Age range	
23–30 31–39 40–49 50+	4 3 3 2
Highest formal educational attained	
Non-literate Primary school Secondary school University	4 4 1 3
Diagnosis	
Depression Schizophrenia Bipolar disorder Alcohol user disorder Epilepsy	1 2 1 1 1
Length living with mental health condition/service	
3–5 6–10 11+	5 5 2

### Participants’ expectation and motivation for involvement

The various reasons for participants’ motivations and expectations were captured using two subthemes: prior experiences, and desire to learn and contribute.

### Prior experiences of involvement

Prior to this study, none of the participants had experiences of active involvement in research. Many of the participants mentioned their experiences of involvement in the capacity building training delivered to prepare them for the current role or the ToC model development/refinement processes leading to this study (P1, P2, P3, P5, P6) as a favorable experience that motivated them to join the current study.

I have never been engaged …except participation in interviews. I had experience of participation only related to this study…to Addis Ababa two times where I shared my experienced on large meeting at big hotel…When coming to this group I was expecting that they have planned to expand that more (previous capacity building training)…I expected that they might have planned to organize service user more so as to achieve our goal… (P2).

At the beginning, some participants had low motivation and were skeptical about the value of being involved, which was explained in relation to lack of prior experiences of active involvement or unpleasant experiences of involvement. However, their motivation gradually improved after getting clarification about the objective, making sense of the relevance of the activities to improve their life and opportunities for active participation. For example, two participants described:

…at the beginning I was not that much motivated and expected something good. I appeared only to sit and see what would happen … I had some concerns in the previous training, which was too long (whole day), very intense and was a bit boring. …[But] as sessions progressed, I realized that the PAR is actually doing about my own problems, we [the group] discussed and worked together something important for people with mental illness…Finally I said to myself “I should actively participate”. (P1)

“I was expecting them (researchers) to ask me questions as they usually do. But this [work] was very different from the previous, we developed a roadmap, established a user organization and developed action plans. I am very happy. Totally different, I get what I did not expect; very interesting thing”. (P6)

### Desire to learn and contribute something valuable

The common thread among many of the participants regarding their key motivation to get involved was wishing to gain more (personal or professional) knowledge and skills that could help them to address issues related to mental health conditions, help others, and address deficits within the healthcare system. Many of the participants expressed emphatically a desire to engage in advocacy and to improve multifaceted injustices experienced by people with mental health conditions (e.g., stigma and discrimination, chaining). Some participants’ motivation was rooted in their own painful lived experiences and observations of how badly people with mental health conditions have been treated at home, within healthcare systems and the community (P2, P3, P6, P7, P9).

…I felt pain when observing many service-users suffering just like I used to. I felt a sort of like the same bad experience. I have been in the same situation for 10 years. Now thanks to God I have passed that bad stage. So I want to contribute. I want to make things better for those suffering with mental illness (P2).

### Experiences of the social dynamics in the PAR process

This theme illustrates how participants experienced and described the active participation, inclusiveness, and respectfulness of the team atmosphere in the PAR process.

### Experience of activeness and inclusiveness of the PAR process

 When asked about personal experiences of and others active participation, and inclusiveness of the PAR process, many participants narrated details of activities they had engaged during the training and ToC development process in previous years (P1, P3, P4, P6). Many of the participants described the process gradually progressed from passive participation at the earlier sessions to more active, and inclusive participatory process overtime as described in the following account.

“…In my group (caregivers), the participation of all individuals was not the same. But we used to let everyone to express idea to his/her level of understanding. Everybody contributed to his/her level of understanding. There was no situation where anyone was passive observer and other individual dominate the discussion…everyone contributed his/her opinion. That was how we used to run the group discussion”… (P1).

Many of the participants appreciated the techniques used to enhance interactive involvement (e.g., small group discussion, reflection sessions) as well as the facilitation process to accommodate all viewpoints by encouraging all participants to share ideas, find solutions, freely interact with each other and make decisions during the PAR process. For example, participants described the process as follows:

"The facilitator…‘operated’ very well to bring all together and discussed in a way that made all open to discuss idea. So my feelings were generally very positive about it [group technique]. I used to anticipate the next session eagerly”. (P3).

“I appreciated… used to encourage high level of involvement of all participants in the PAR process… accommodated everyone to have chance to express opinion. I think everybody got a time to reflect opinions. That is one of the reasons I committed and stay engaged in the PAR process”. (P5).

Many participants spoke about the logistical practicalities, including the convenience of the time and place of the PAR sessions, and financial compensation for their time (P1, P2, P10, P3, P11) as key factors for their active participation.

“… We utilized our time efficiently; because we come on time, engage in the discussion effectively and complete on time…because we scheduled it [the meetings]during public holidays (out of our working time). We used to meet two days per week for two to three hours, which was very easy contribution of time”. (P1).

The experience of active inclusiveness of the PAR process was not homogeneously reported by all participants. For example, one participant noted that: “*there was one participant who did not say anything*” P3,and expressed concern that potentially articulate participants were not recruited. Some participants expressed some challenges encountered in group activities that involved writing and reading because of their low literacy (P2, P6, P7,P10). However, they appreciated the oral reflection and use of various techniques that helped them to get adequately involved.

…our involvement is very active…although that was challenging to me when that required doing it in writing…although we cannot write we effectively contributed in the discussion and generating ideas (P7).

### RPG perceived outcomes/benefits of the PAR process

Linked to their involvement in the PAR process, the participants described experiences of positive outcomes/benefits at personal, social, research and healthcare service.

### Personal benefits

All the participants made positive comments and reported personally gaining from being part of the PAR process in terms of improved knowledge and skills, self-confidence, health, and feeling of achievement and contribution.

 Almost all participants spoke of gaining knowledge about mental illness, treatment, and about managing mental illness during PAR sessions from each other, and education delivered by professionals. Some participants particularly appreciated the discussions with RPG members outside of the clinical context. The value of critical reflection instilled into the PAR process enabled them to uncover issues which had received inadequate attention and gain a deeper understanding of service users` expectations, unmet needs and the gaps in the healthcare service and their own gaps (e.g., inadequacy of information about medication use, lack of focus on physical health).

"There was a lot of education in the group…we discussed ideas, freely exchanged ideas in small groups. We exchanged education from the facilitators and our presentations…The education works for service users… We can identify people chained at home through education….I got additional knowledge…. Now I can teach my neighbors during coffee ceremony and at work place. I advised them to go to healthcare service and take medicine and medicine can help for recovery. Now I am teaching how to safely use medicine”. (P10).

Some participants spoke about improved communication skills (writing and public speaking) through the writing and reflection during the group sessions, public dissemination and public speaking, and developing research skills that could be immediately applied in their daily lives or professional development.

The PAR process taught me a lot. First, I noticed that I can generate useful ideas from beginning to the end of all sessions; there were several thought provoking ideas discussed during the research process that creates ‘Ha!’ here am correct and I have created sense of being of value. Second, I have gained knowledge about how to develop action plan; how to start planning, with whom to work, about sources of support…HA! … This is not only for the study, but it is very important for personal life. I have learned how to live planned in my personal life. (P3)

Some participants described health related benefits such as improvements in their mental health or healthcare habits/coping mechanisms and improved social life. For example, some participants reported increased medication adherence and visiting healthcare service more often (P2, P9), reducing (P1, P9) or totally stopping (P4) alcohol consumption, observed others stopped drinking alcohol while taking medication (P1), improved self-and others care (P2,P7, P10), reduced family (P4) or neighbors disturbance (P7). Some participants mentioned that participation in PAR process offered them a platform to exchange experiences with people who have similar mental health conditions, which helped them in terms of reduced feelings of depressed, worries or anxiety (P2,P6), combating loneliness (P6,P7, P8,P9), improved satisfaction with life and hope (P2, P6,P7, P9), being able to comfortably talk about mental illness (P2,P6, P9).

I have developed the confidence to take medication without fear of side effects. I am taking advice from health professionals about all side effects…I have great improvement after getting this experience in many aspects of my life. I have improvement. I am happy. I have hope. Thanks to God now I am health. I have recovered in health… I am relaxed… Thanks to God…I have passed that difficult/crisis time, that dark time; now I am in light… (P2).

The participants reported that involvement in the PAR processes positively influenced them in terms of improved self-confidence, which was evident in some participant comments about the personal changes in their capacity to communicate in situations outside of the group sessions:

…That helped her very much. She had no training before and lacked knowledge; after the training and involvement in the research she gets improved much more. For example, before she did not speak more in public, now she started expressing herself very well in group discussion; … I saw her asking and speaking in social association(Idir)…,during coffee ceremony at home and neighborhoods she started teaching about the causes and problems of mental illness. … (P1).

All participants reported that the PAR process was a valuable investment of their time working together to address issues of relevance to themselves and that directly impacted on mental health service improvement. The participants mentioned the action plan developed to raise public awareness, the establishment of service user association, and to empower the association as their valuable contribution, which they expressed as sense of achievement, contribution, and agency:

“…We become united… because of the participation in the research group we have got freedom, now we are organized as service user association…we started saving money in bank…we started saving that can strengthen our relationship. I am very happy”. (P2)

“…One of the hopeful gains is the established service user association. More than what we put in our stomach (eat) and pocket, better to support this hopeful organization. We are expecting the licensing of the service user association”. (P7)

### Social benefits

 The participants mentioned the opportunity to meet other people, combating loneliness and engagement in important activities and improved social acceptance as key social benefits of their involvement in the PAR process. Some participants expressed their experiences of living alone or having very limited opportunity to get out of home to interact with other people outside of their family circles (P2, P7, P8, P9). Some placed high value on spending time together and the positive experiences of the informal interaction and laughing (energizer in-between sessions), sharing ideas and experiences during the PAR sessions. Some participants explained the value of the PAR process looking back to the painful experiences of feeling ignored by people who were close acquaintances, including family members.

“…I consider participation in this group as my rebirth. This is the chance I missed in my entire life. … I have been a person discriminated and neglected for a lifetime…Now other people started appreciating the improvements observed in myself; they are saying to me “you are really getting young”. They say “she becomes new person… improved” talking at my back. When I hear this, I feel deep satisfaction… I keep my hygiene, dress very well and enjoy with my children. I dress my hair well and just I am free. Thanks to God…This is new beginning of my life…” (P2).

“…This helped me to improve my relation and communication with people; the value people give has improved. This makes me happy… I developed skills of participation with people, sitting with others and working with others. I used to pass time locked up at home; passing time here is very interesting. This is leisure time and recreational…there was no such opportunity…this helped me not to remain hidden or neglected. Here I have freedom to share my ideas. I have nice times here…”. (P8)

Some participants (P2, P3, P4, P6, P7) spoke about social acceptance and improvement in public attitude towards them.

“…I have received feedback from different people. I have presented the finding of the research to representatives of different community and government offices. Our findings touch every sector, gender office, health office, psychiatry and others. People appreciated my presentation and commented that I was unnecessarily quiet for too long. There were good things”. (P3)

### Benefit to research and the health care system

 When asked about what their involvement in PAR benefit or potentially contributed to healthcare improvement, the participants mentioned many examples of activities during and after the PAR process that could improve mental health services. For example, some participants reported volunteering in various activities, including giving support to people with mental health conditions (peer support), e.g., finding cases and supporting them to access healthcare (P1, P2, P7), and giving education and advice (P1, P3, P7, P10).

“…In addition to the lesson I get here, I shared a lot from my lived experience to change and motivate people to take their medication properly. In my neighborhood I have supported many people to take medication properly and told them that they could recover through proper use of medicine. Even if the patients resist taking the medicine, caregivers need to help with the medication [of a family member] through negotiation. For many people, I personally, gave education. I have tried all my best”. (P10)

When asked about any improvements in the hospital or healthcare centers after their involvement in the PAR process, some participants who had chances to visit the health facilities expressed their observation in improved receptiveness and healthcare service delivery.

“…We have clearly discussed with health professionals about their problem in patient care, receptiveness, medication availability and …we discussed many issues. After I got involved in the research I had visits to the hospital for another individual and for my child, and I noticed that they are doing well…There is some improvement that can be appreciated”. (P6)

The health professional participants also spoke about readiness to apply the knowledge and skills they gained from the PAR process to improve the healthcare service delivery (e.g., the way they diagnose and treat patients, provision of sufficient information).

“…Before involvement in the research, health professionals had self-distancing or pushing behavior towards mental health service users. We (health professionals) used to say the psychiatric nurse's (…named) people came (mental service users)… consider only the psychiatric nurse in charge of the mental health service. More recently we are working with him at the psychiatric unit. This collaborative work needs to be strengthened”. (P5)

Some participants (P1, P3, P5) mentioned that the increased involvement and collaboration of the diverse local stakeholders’, political officials` willingness to undertake empowering actions for service-user involvement and promises to solve problem of availability of medication could be an important initiative towards improving the mental health service.

“… If we are able to work sustainably with these stakeholders including religious institutions, schools teachers and students we can bring change. The community stakeholders who participated in the workshop can support and build capacity. The hospital management body, including the medical director and CEO participated in the final stakeholder consultative meeting and discussion on research participant group findings’ dissemination. They have agreed to integrate the research participant group in the health education mainstream routine of the hospital”. (P5)

“…There is some change; now a roadmap has been developed with stakeholders. A range of stakeholders that included religious leaders, education sectors, community associations (Idir), health professionals and all others received some training. Now, there is good beginning. (P1)

### Implementation challenges and recommendations

 When asked about challenges experienced during the PAR process, most participants gave neutral responses, e.g.,  ‘no problem ‘or ‘everything is ok, or no need for modification’ and only few participants (P1, P3, P5, P6, P7) made comments about ways to improve the study, which focused on the need to cover wider healthcare areas and involve more participants, and enhance support for the service user association at follow up.

 However, when asked about what support they would need for strengthening service-user involvement, the participants mentioned various logistics, funding, systemic and organizational constraints to be resolved. As an immediate challenge, the participants mentioned logistical problems (meeting places/office). The participants also stated the need for financial support to cover engagement related costs, including time compensation and transport costs for members who could be involved in future PAR activities, as well as the need for more capacity building and training materials for community awareness-raising to pursue their next action plans.

All participants had deep concern regarding the long time taken by district officials to reach a decision about licensing/registration of the new service user association. Some participants also noted that mental healthcare was not effectively integrated within the healthcare/hospital settings, where there are general health workers trained in first line mental health care.

“I repeatedly visited the relevant administration office in the district to facilitate the registration of the association. But it is still challenging and took a lot of time for them [administrators] to respond and endorse the registration of the service user association. There was little practical support and I have completely giving up hope. I find that they are not moving as they promised during the workshop”. (P4)

“There is a tendency to push the healthcare service for people with mental health conditions to the psychiatric nurse alone. There are trained professionals in mhGAP, but are not appointed to the psychiatric service unit…the system has been working as it was for years… (P5).

The participants (P1, P4, P6, P7) suggested for expanding the scope of the study, more collaborative working at different levels within the healthcare system, enhancing stakeholder collaboration, community mobilization and addressing mental health medication problems to support the initiative sustainably.

“The current study was a bit narrow. First, it seems it had limited budget to involve more people over longer time. This is one important area that needs improvement. Secondly, the political leader should make mental illness a mainstream routine activity. Unless mental illness was supported with political leaders and government, this research group alone with external assistance… may not last long …and be able to bring sustainable change…Government has to put their hand in this initiative… Therefore, as ownership to these initiatives our local government needs to give attention, allocate budget, take it as mainstream agenda, otherwise I do not think this would be sustainable…the involvement of political leaders in this initiative need to improve”…(P1).

## Discussion

In this study, we described the process and experiences of involvement in a pilot model of service-user involvement using PAR in rural Ethiopia. This study is one of only a few empirical studies of service-user involvement in mental health systems in LMICs. The PAR approach demonstrated potential to achieve equitable and diverse stakeholder involvement, establishing a partnership, and recognizing the value of service-user contributions. The findings highlighted the importance of multiple consultative meetings, building capacity and collaborative working with diverse stakeholders in empowering service-user involvement. Participants valued the process of PAR and identified a range of benefits, particularly feeling equipped with knowledge and included within society. This study identified some structural challenges to embedding service-user involvement in the mental health system in Ethiopia.

### Creating an inclusive participatory space

The pilot process created a communicative space; a platform in time and space where diverse stakeholder groups could come together and enter into constructive dialogue to share, understand and change key points of common concern to them [12, 28]. We employed various strategies to create the communicative space. First, our study was built on existing community strengths, collaborative working experiences, and infrastructure, which was evidenced by diverse stakeholder involvement experiences during our formative studies [8, 9] and studies in relation to a previous project seeking to expand access to mental health care locally [19]. In line with existing evidence, building on pre-existing relationships provided valuable opportunities to build trust and optimize engagement [29].

Second, in line with our proposed key interventions from theToC, towards strengthening service-user involvement through community collaborative engagement [9, 12], we set up two multi-stakeholder groups (RPG and RAG), conducted capacity-building through training and workshops to enhance formal community participation, collaborative structures, and equip participants for active partnership. Existing evidence supports the importance of capacity building for key stakeholders, forming trusting relationships and alliances with them as a key mechanism for active involvement, challenging the barriers that marginalize service-user involvement, and promoting shared understanding of service-user engagement activities [1, 6, 29, 30].

Third, our findings show that application of participatory techniques, e.g. small group discussions and critical reflection, and prioritization exercises can enhance creation of an active inclusive participatory space. These techniques can provide all stakeholders with equitable opportunities, and uniquely provides opportunities for those who are marginalized (in our case service-users) to participate as equals to voice their views, thus reducing the risk of tokenism [31]. The critical aspect of PAR meant that we used a variety of direct and indirect questions to initiate critical dialogue among stakeholder groups, clarify key gaps in the health system and set out priorities for action. For example, the stakeholders were openly critical of the healthcare system for failings related to mental health care (e.g., inconsistent supply/lack of psychotropic drugs, low budget, and lack of trained mental health professionals). This finding accords with several PAR studies that advocate to provoke participants to surface their values, beliefs, and purposefully employ strategies to interrupt and challenge social, cultural and political structures and practices, which marginalize service-users roles, which results in new forms of relationships and more informed ways to deal with problems [28, 31]. The apparent success of these approaches was particularly encouraging given the pre-PAR reluctance of service users to express their views [9].

### PAR process outcomes

Our findings indicated examples of the added value of PAR towards intermediate outcomes that were expected within the ToC model [9], including enhanced inclusive participation, improved stakeholder collaboration, a range of personal and social benefits for RPG and lessons that could help to refine the ToC model to increase impact. Specific examples of these outcomes in our study are discussed here.

Our findings showed that the active involvement of diverse stakeholder groups in PAR enhanced willingness to work in collaboration, commitment to mobilize resources and prompted several course of actions to overcome some challenges to service-user involvement using their local capacities. Such local mobilization included initiating financial contributions, promising to create mechanisms to generate income, and establishing a community advisory committee to support the service user association movements in awareness creation, advocacy and lobbying for resource mobilization. Our finding also shows the feasibility and acceptability of what has been considered imperative and anticipated early during our ToC development about the need to establish a multi-stakeholder response and collaborative working as crucial contact points for increasing service-user involvement [9].

 The participants (RPG) described numerous personal benefits from being involved in the PAR process, including gaining knowledge and skills that helped them to take action to improve their living conditions and situation at home/work, improved confidence and more positive sense of self, improved communication skills (public speaking). Many of them also mentioned experiences of social benefits in terms of feeling ‘free’, having the chance to work with others, building friendships and trusting relationships. These experiences and benefits were in line with what were anticipated as short and medium term outcomes in our ToC model [9]. Evidence from small scale, largely retrospective accounts of engagement in PAR studies, predominantly in high-income countries, found similar multi-dimensional impacts of involvement in PAR, e.g. meaningful social support, and sense of empowerment because of the critical dialogue and reflection embedded in the PAR process that improves critical consciousness about sources of oppression and capabilities to deal with a problem that is identified by the participants themselves [31, 32]. The service-user participants also reported experiences of improved public acceptance, attitudes and opportunities for engagement in social activities in their local community. This is in line with the evidence showing that close, targeted, and continuous positive social contact between service-users, health professional and other key health system stakeholder are effective strategies to foster mutual understanding, social inclusion and reduce stigmatizing attitudes [33, 34].

Another important outcome of this study is establishment of a service user association in Sodo district for the first time. The need to organize and empower the association has been a priority in the study area [8], and was also one of the key intermediate outcomes identified in the ToC model [9]. Evidence shows that service user associations can make a variety of contributions to healthcare system inprovement, including education and advocacy to raise the public health priority of mental health [6], pooling of funds for medication, peer support, social inclusion, promoting recovery and personal agency, and advocating forthe protection of service-users’ rights [35, 36].

The application of the PAR approach was important for the pilot implementation of the ToC model in many ways. As well as promoting inclusive active involvement and collaboration of diverse stakeholder groups, the PAR prioritization exercise ensured a shared understanding of local priorities and target areas that are likely to bringthe greatest potential benefits in the local context [37].This allowed us to make the generic ToC contextually specific [9]. In the same vein, the PAR process showed the availability and the importance of tapping into rich community resources and assets such as social institutions, e.g. religious and faith-based organizations and schools, as key partners in awareness-raising and improvement of mental health services. Evidence in our study setting [38, 39] and many LMICs show the abundant availability and considerable reliance on traditional and religious/faith- based healers as as contributors to mental health cre [40]. This is in line with the strength-based principle of PAR [41], which emphasis the need to work and bring together multiple perspectives, resources, skills, shared leadership to address intersecting factors (e.g., social, environmental, economic, political) greater emphasis on institutional/political and social responsibilities for removing barriers to disadvantaged groups to use their capabilities and address social injustices [42].

Third, our study also showed that the micro- and meso-level interventions alone were not enough to implement service-user involvement in improving the primary mental healthcare system. Rather intervention pathways need to extend to the strategic level. Scholars in the area also recommend the need to include a broader focus on ToC, even if problems are systemic and distal (e.g., poverty, social inequality)in nature, because doing so helps to promote recognition of the multi-factorial nature of social or health needs and to recognize the intervention in its systemic context and to see it as one part of wider picture of impetus for change [10, 43, 44].

### Challenges to embed service-user involvement in primary healthcare

Our findings show key structural issues that demand attention for service-user involvement to become a routine, normalized way of working in the primary mental healthcare system. Many of these barriers fall somewhat outside the scope of the current study, but negatively affected our attempt to legitimately embed service-user involvement. Some of these challenges could be addressed with available resources and drawing on the readiness of the local stakeholders, but putting this into action is still in its infancy and needs more follow up and support for resource mobilization. These challenges have been well documented in the literature as key factors that need to be addressed for effective implementation of service-user involvement [1, 2].

Our study was conducted in a context with intersecting health system challenges such as budget limitations, inconsistent supply of medication, low socio-economic status of service-users (e.g., unemployment, poverty, low education), and pervasive stigma and discrimination [45, 46]. More importantly, there is no national regulatory mechanism to enforce planners and healthcare workers to involve service-users in mental health system strengthening in Ethiopia and involvement has not been explicitly stated in strategic health policy documents [8]. There is no officially approved mental health legislation [47], which is an important mechanism for protection of human rights of marginalized peoples and necessary to ensure appropriate, adequate, timely, and humane health-care services [4]. These deficits had a negative impact on efforts to embed service-user involvement. In our case for example, although district officials were interested to establish the service user association and were actively involved in the PAR process; later we noticed that they struggled to overcome bureaucratic barriers to make it happen. Effective integration of service-user involvement in mental health system demands creating national structures(e.g., policy contexts), cultures (governance and organization context) and practices, as well as local level structures to ensure accountability [1, 48].

We believe that mobilizing and empowering service user associations at grassroots to advocate for themselves can create social movements that compel strategic level decision-makers to acknowledge the reality of service-users and achieve structural changes (e.g., influence policies) and protect the rights of service-users [35, 49]. In addition, a rights-based approach to service-user involvement that emphasizes redressing the unfair distribution of power, economic, social, and cultural rights, discriminatory practices that hinder service-users realization of rights is needed [50].

## Strengths and limitations

Our study is one of only a few studies to apply an inclusive participatory approach to implement a complex model of service-user involvement in a low-income country setting. The active involvement of stakeholders at all stages of the study increases the authenticity and trustworthiness of the findings. In addition, our study drew on multiple methods and sources of data that provided greater depth to capture comprehensive accounts of the processes and experiences of involvement. The prospective nature of the study and observational data on the implementation process is a key strength, which is an important part of case study [51]. The researcher(s)’prolonged involvement with stakeholders allowed the participants to be more reflective of their true feelings towards their participation.

However, the findings of this study need to be interpreted within the context of its limitations. Power dynamics affecting service-user involvement were stillobserved to be a challenge, even with our efforts to achieve a majority of service-users in all collective meetings and facilitated sessions so that service-users would feel comfortable in expressing their views. It was very difficult to involve the participants in interview data analysis, due to the participants being either illiterate or low literacy. Our study did not include more strategic level stakeholders due to time and resource constraints. This study was small scale, based on a single case study with no comparative case or control group. thus limiting representativeness of all experiences of service-user involvement or generalization that can be made. Hence, a study of wider scope might be needed that could enrich future service-user involvement models. However, we believe that our findings can provide valuable insights about what is needed to support successful collaborative working with stakeholders to achieve mental health service user involvement in similar settings.

PAR needs time, however, we were constrained to a short implementation time that limited our ability to achieve implementation of action plans; the action stage was underdeveloped and implemented part of our study. Hence, the findings need to be considered preliminary; we recommend additional follow-up commitment to take action on the priorities identified. Further refinement of the model with broader evidence synthesis and testing in wider contexts would ensure usefulness of the model to replicate in wider areas.

## Conclusions

Our case study shows that the application of the PAR approach can offer a useful, feasible, and acceptable mechanism to implement a complex model of service-user involvement in a resource constrained setting. Building trusting relationships with community stakeholders, the willingness of leaders, and existing structures and community networks were crucial for the success of this study. Given the multilevel discrimination and stigma against people with mental health conditions, we believed that further critical dialogue is needed to challenge the status quo and achieve action at higher policy levels to further enable the involvement of people with lived experiences within the mental health system.

## Supplementary Information

**Additional file 1. **Quality criteria.

**Additional file 2. **Summary of stages, activities, and session plans for the PAR process.

**Additional file 3.** Summary of consultative workshop aims andactivities.

**Additional file 4.** List of top tenpriorities by stakeholder groups.

**Additional file** **5. **Themes, subthemes and illustrative quotes.

## Data Availability

All data generated or analysed for this study are included in this published article (and its supplementary information files).

## Abbreviations

CAB: Community Advisory Board
CST: Critical social theory
Emerald: Emerging mental health systems in low- and middle-income countries
HEWs: Health extension workers
LMICs: Low- and middle-income countries
NGT: Nominal group technique
PAR: Participatory action research
PRIME: PRogramme for Improving Mental health carE
RAG: Research Advisory Groups
RPG: Research Participant Groups
ToC: Theory of change
