# Supplementary material for: Using participatory action research to pilot a model of service user and caregiver involvement in mental health system strengthening in Ethiopian primary healthcare: a case study
Source: Int J Ment Health Syst. 2022 Jul 11;16:33. doi: 10.1186/s13033-022-00545-8 (PMC9275138; doi:10.1186/s13033-022-00545-8)
Supplement: Supplementary file 1 — Additional file 1. Quality criteria. [file 13033_2022_545_MOESM1_ESM.docx]

Additional file 1. Quality criteria

| Quality criteria | Description |
| --- | --- |
| Credibility | The research was implemented within pre-established community participatory structures (e.g. PRIME). In addition, the facilitator of the PAR process (SA) had engaged for an extended period in the study area whereby he presented findings of formative research, facilitated all the workshops and PAR sessions with diverse stakeholders. This provided an opportunity to build authentic, trusting rapport between SA and participants ([1](#_ENREF_1)). SA had a background in psychology and social work with training and experience in PAR, had conducted systematic reviews about PAR and attended several online training courses that helped him to facilitate the PAR process ([1](#_ENREF_1), [2](#_ENREF_2)). During data collection, the interviewer summarized the participants’ responses throughout the interviews to confirm participants’ viewpoints that can serve as member checking. |
| Consistency /construct validity | During the extended period in the field, multiple sources and methods of data collection including recordings of workshops and PAR sessions, minutes, flipcharts and oral presentations, participatory observation and in-depth interviews were utilized as described in the data collection section, which helped for triangulation ([3](#_ENREF_3)). Iterative reflection and reflexivity in a sustained dialogue with participants helped as member checking of the data ([1](#_ENREF_1), [2](#_ENREF_2)). |
| Transferability | We have provided in-depth, detailed description of the study setting and activities of at all stages of the PAR process by publishing a study protocol ([4](#_ENREF_4)). Furthermore, we provided in-depth, detailed and descriptive analysis of the data using the participants perspectives, proper documentation of data, activities, and thick descriptions by quoting participants responses to substantiate the findings as found in the results section and supporting with supplementary files ([1](#_ENREF_1), [3](#_ENREF_3)). |
| Dependability | We gave thick/rich description of the study process through transcripts and field notes written by SA to record his reflections and experiences and consider any emerging issues in the data throughout the analysis ([1](#_ENREF_1)), have conducted reflection with stakeholder during each PAR session and presented preliminary process findings to community stakeholders in consultative workshop; and the senior co-authors reviewed(CH and HL) the analysis process of the paper ([3](#_ENREF_3)). More importantly, the protocol to this study was peer reviewed and published ([4](#_ENREF_4)). |
| Conformability | The stakeholder groups (particularly RPG) were involved in all stages of the PAR process whereby they maintained reflexivity and discussed on the summary of each session. Two participants (SA and a colleague) reviewed written summaries of the results in relation to themes and subthemes with which their original data were linked. Furthermore, the preliminary findings of the PAR were presented to stakeholder groups for review and validation of the process findings. This was also ensured by audio recording of group discussion. In addition, each emerging subthemes in the findings were substantiated with rich quotes extracted from the participants responses ([1](#_ENREF_1)). |
| Catalytic validity or authenticity also called Tactical authenticity or Educative authenticity or emphatic validity | The stakeholder participants were provided with a detailed presentation about mental health services, the situation of service-users and the barriers and facilitators to service-user involvement in the local context. This helped the participants to become familiarized with the realities of service-users, prioritize action areas and generate action strategies, which is noble knowledge production with new possibilities for social action ([2](#_ENREF_2), [5](#_ENREF_5)). Health professionals, caregivers and service-users were trained to involve in mental health system strengthening ([5](#_ENREF_5)). The PAR process also helped RPG members to understand each other as well as reported feelings of empowered ([2](#_ENREF_2), [5](#_ENREF_5)) |
| Outcome validity | The findings reported in the case study were the outcomes of a series of reflective cycles with stakeholders. The stakeholder groups identified top priorities for action, has started awareness raising intervention, community resource mobilization to empower service-user groups and agreed to mainstream service-user support in local statutory formal routines and community structures ([5](#_ENREF_5)). |
| Democratic validity also related to: local validity, relevance or applicability, ecological validity/participatory validity | The findings of this study were co-produced in collaboration with stakeholders who involved with their consent voluntarily whereby they identified locally relevant priorities and solutions ([2](#_ENREF_2), [5](#_ENREF_5)). Moreover, the voices of all participated were accommodated using systematic approaches as detailed in our study protocol ([4](#_ENREF_4)). |
| Dialogic validity | This study applied PAR approach to pilot study that enables equitable and inclusive participation of all stakeholders including service-users. The case study reported here was the outcome of multiple sources and methods data collection and documentations ([5](#_ENREF_5)). The iterative cyclical PAR process and the critical and reflective dialogue with stakeholders helped to strength local stakeholder collaboration, enhanced working together and understand top priorities for action to empower service-user involvement in mental health system improvement ([5](#_ENREF_5)). |
| Process validity | Various triangulation mechanisms have been used throughout the study through participatory observation of group dynamics, accounts of each participants, challenges encountered and data generated in the three stages of the PAR process through a series of iterative reflection([5](#_ENREF_5)). |
| Communicative validation | The research was carried out in collaboration and active participation with community stakeholders and also with the authors team that enabled as auditors and critics of the process ([5](#_ENREF_5)) |
| Ethical validity/fairness | A range of perspectives from diverse stakeholder was considered. The participants entered the study fully informed, reflected through the PAR process that they are doing something helpful to them and improve mental health system. They expressed satisfaction with all voices equitable participation and humanly treatment ([1](#_ENREF_1), [2](#_ENREF_2)) |

References

1. Lincoln YS, Lynham SA, Guba EG. Paradigmatic controversies, contradictions, and emerging confluences, revisited. The Sage handbook of qualitative research. 2011;4:97-128.

2. Springett J, Atkey K, Kongats K, Zulla R, Wilkins E, editors. Conceptualizing quality in participatory health research: A phenomenographic inquiry. Forum Qualitative Sozialforschung/Forum: Qualitative Social Research; 2016.

3. Yin RK. Case study research: design and methods 5th ed. Thousand Oaks. 2014.

4. Abayneh S, Lempp H, Hanlon C. Participatory action research to pilot a model of mental health service user involvement in an Ethiopian rural primary healthcare setting: study protocol. Research Involvement and Engagement. 2020;6(1):1-14.

5. Herr K, Anderson GL. The action research dissertation: A guide for students and faculty: Sage publications; 2014.
