# Supplementary material for: Using participatory action research to pilot a model of service user and caregiver involvement in mental health system strengthening in Ethiopian primary healthcare: a case study
Source: Int J Ment Health Syst. 2022 Jul 11;16:33. doi: 10.1186/s13033-022-00545-8 (PMC9275138; doi:10.1186/s13033-022-00545-8)
Supplement: Supplementary file 2 — Additional file 2. Summary of stages, activities, and session plans for the PAR process. [file 13033_2022_545_MOESM2_ESM.docx]

Additional file 2

Summary of stages, activities, and session plans for the PAR process.

| Stages | Cyclical activities | Descriptions | Time periods |
| --- | --- | --- | --- |
| Stage1: **Establish stakeholders groups, capacity building training /consultative Workshop and prioritization exercise** | Plan | Identify and establish cross-stakeholder groups that services as a reference group, and working group together with Sodo district health office  Getting stakeholder groups and agree on time and place for regular sessions  Develop summary of findings from foundational studies  Identify and prioritize top thematic concerns | Between March 2018-August 2019 |
|  | Act | Conduct capacity building training and consultative workshops, present and discuss on foundational studies in a consultative workshop with stakeholder groups  Systematically identified priority areas through small homogenous groups and heterogeneous group discussions, prioritize top areas using Nominal Group Techniques | Between March 2018-August 2019 |
|  | Observe | Conducted pre-post training survey about perception of training, knowledge and attitude as well as exist and follow up in-depth interviews,  Collected key priorities generated in small group and plenary sessions, through audio-recording, captured minutes, field notes  Principal investigator/a research assistant recorded field notes on group dynamics and interactions and on the context surrounding the discussion. | Between March 2018-August 2019 |
|  | Reflect | Discussed on and reached consensus on ways forward and priority areas  Reflected within homogeneous groups, heterogeneous; compare the reports of each group  The stakeholder groups make sense of what has happed through thinking about how it fits with their experiences and local contexts using criteria | Between March 2018-August 2019 |
| **Stage2: Development of program and action plan** | Plan | Reached common understanding between RPG and the researchers and assistants what the research involves and ensure consent to participate  RPG agreed on time, place, number of sessions per week and duration of the sessions at primary health facility level(Buie Primary Hospital)  Reviewed the thematic priorities identified in Stage 1, discussed, selected and prioritized one priority for action as trial of proof of concepts  Generated set of solutions and design intervention strategies | Between August 2019-December 2019 |
|  | Act | The RPG identified gaps in lack of awareness, available community resources and assets, target partners, and developed implementation strategies and action plans | Between August 2019-December 2019 |
|  | Observing | SA conducted participatory observation and documented the process through notes, and audio-recordings, summaries of discussions on flipcharts and photographs  Evaluate participation and representation | Between August 2019-December 2019 |
|  | Reflection | RPG conducted continuous reflection throughout the action planning stages on data from observation, field notes and reflect on the action options  Examined whether the proposed improvement methods was feasible in terms, time, additional resources availability, and local experiences | Between August 2019-December 2019 |
| **Stage3:Preliminary implementation and evaluation** | Plan | RPG reviewed the developed program and action plans and reach agreement about the way strategies would be put into operation and how to document observations  RPG designed implementation and action strategies | December 2019 |
|  | Act | Conducted consultative workshop with multi-stakeholders  Disseminated preliminary process finings from Stages 1-2  Conducted awareness-raising education and experiences sharing for stakeholders  Established community advisory committee to support service user association  Reached consensus with stakeholders to sustain the activities of the research in their local contexts on their own | December 2019 |
|  | Observe | SA documented the consultative workshop process through taking detailed field notes, observation and discussion with RPG and community stakeholders  Preliminary analysis and findings of the process will be collected  Conducted in-depth interviews with RPG to ascertain their perceptions and experiences of the process of PAR | December 2019-January 2020 |
|  | Reflect | Conducted evaluation meeting with RPG and community stakeholders and collect feedback about the process of the PAR process, and reflected on the process of implementation  RPG members identified options to sustain the PAR and action with minimal or without academic researchers | December 2019-January 2020 |
