# Supplementary material for: Using participatory action research to pilot a model of service user and caregiver involvement in mental health system strengthening in Ethiopian primary healthcare: a case study
Source: Int J Ment Health Syst. 2022 Jul 11;16:33. doi: 10.1186/s13033-022-00545-8 (PMC9275138; doi:10.1186/s13033-022-00545-8)
Supplement: Supplementary file 3 — Additional file 3. Summary of consultative workshop aims andactivities. [file 13033_2022_545_MOESM3_ESM.docx]

Additional file 3. Summary of consultative workshop aims and activities

| Training/  Workshop | Aims | Activities |
| --- | --- | --- |
| March 2018 | To ensure service users, and health professionals/managers have the necessary knowledge and skills for active participation and working collaboratively within mental health systems | Two training manuals were produced([60](#_ENREF_60)) and provided to 12 service users, 12 caregivers and 18 health professionals  The training for health professionals/managers (n=18) was conducted for a full day (7 hours).The initial training for service users and caregivers (n=24) was three full days, followed by seven biweekly photovoice sessions of 3-4 hours duration. |
| Workshop 1(August 2018) | Generally to learn the situation of service user involvement in mental health systems strengthening in the study site in relation to the global situation, specifically to:  Create awareness about international experiences of stakeholders’ roles in relation to mobilization and empowerment of service user association, and the roles of service user associations in mental health systems strengthening  Create mechanisms to gauge stakeholder groups’ interest, collaboration, and sense of ownership of service user involvement in mental health systems strengthening in their local context;  Discuss, and seek stakeholders feedback about the draft bylaws of the service user association; and  Establish mechanisms for formalization of the certification of the service user association and link with the local community. | Two co-authors (AA and SA) with the facilitation of the then district administration head, presented on discussed on:  Existing experiences on the role of service user organizations in mental health system  Role of stakeholders in empowering and spotting windows of opportunity in the local stetting service user involvement  Draft bylaws for service-user organization |
| Workshop 2(August 2019) | To create awareness about what is known about the facilitators and barriers to service-user involvement in the local context,  Discuss on ways to tap local community resources and assets to empower service-user involvement in mental health system strengthening  To reflect on the ToC map and identify top priorities for action to effectively involve service-user in mental health system strengthening | First author (SA) and research assistant (a PhD student) with facilitation of the then district administration health office head presented and discussed on formative studies in the area on:  (i)Involvement experience, barriers, facilitator and capacity building for service-user involvement([17](#_ENREF_17)), (ii) components of the co-produced ToCmodel([18](#_ENREF_18)), systematic review about the application of PAR for service-user involvement in mental health system(unpublished), (iv) two relevant studies about local community resources and assets ([61](#_ENREF_61), [62](#_ENREF_62)) with potential for use to empower service-user for involvement in mental health strengthening |
| Workshop3 (December 2019) | To foster stakeholder collaboration and support through dissemination of preliminary findings of the PAR process, and awareness creation to diverse stakeholder groups | Four key activities were accomplished: Members of RPG presented highlights of ToC, prioritization exercise outcomes and details of the process findings of the Stage to PAR processes on program and action plan to a broader range stakeholder groups  Conducted awareness-raising education to enhance stakeholders’ awareness about mental illness and roles of service users within mental health system strengthening(By a senior professor of psychiatry (AA) from Addis Ababa University)  Three people from two service-user associations (Mental Health Users Association in Ethiopia-from Addis Ababa, and Sodo district Association of People Living with HIV/AIDS) shared their lived experiences, association, and how to collaboratively work in empowering service user association.  RPG convened discussion session for stakeholders to reflect on the presentation, and develop collective views on action plans for next steps and ways forward |
