# Supplementary material for: Using participatory action research to pilot a model of service user and caregiver involvement in mental health system strengthening in Ethiopian primary healthcare: a case study
Source: Int J Ment Health Syst. 2022 Jul 11;16:33. doi: 10.1186/s13033-022-00545-8 (PMC9275138; doi:10.1186/s13033-022-00545-8)
Supplement: Supplementary file 4 — Additional file 4. List of top tenpriorities by stakeholder groups. [file 13033_2022_545_MOESM4_ESM.docx]

Additional file 4

Table S1. List of top ten priorities by stakeholder groups

| No | Topic | Maximum value assigned by stakeholder groups | | | | |
| --- | --- | --- | --- | --- | --- | --- |
|  |  | Service user | Caregivers | Health professionals | Leaders | Mean value |
| 1 | Stigma and discrimination | 7 | 10 | 7 | 8 | 8 |
| 2 | Low service user and caregiver involvement | 5 | 4 | 6 | 3 | 4.5 |
| 3 | Socio-economic problems | 6 | 6 | 4 | 6 | 5.5 |
| 4 | Lack of stakeholders collaboration | 2 | 2 | 8 | 5 | 4.25 |
| 5 | Inadequate and inconsistent supply, problem of use of Psychotropic | 10 | 8 | 5 | 9 | 8 |
| 6 | Multilevel lack of awareness about mental illness | 9 | 9 | 10 | 10 | 9.5 |
| 7 | Communication problem(at home and with health problems) | 4 | 5 | 2 | 1 | 3 |
| 8 | Lack of organization of service users | 8 | 7 | 1 | 7 | 5.75 |
| 9 | Lack of rehabilitation center | 3 | 3 | 3 | 2 | 2.75 |
| 10 | Risk factors for mental illness(Khat and alcohol use) | 1 | 1 | 9 | 4 | 3.75 |
