# Supplementary material for: Using participatory action research to pilot a model of service user and caregiver involvement in mental health system strengthening in Ethiopian primary healthcare: a case study
Source: Int J Ment Health Syst. 2022 Jul 11;16:33. doi: 10.1186/s13033-022-00545-8 (PMC9275138; doi:10.1186/s13033-022-00545-8)
Supplement: Supplementary file 5 — Additional file 5. Themes, subthemes and illustrative quotes. [file 13033_2022_545_MOESM5_ESM.docx]

Additional file 5. Themes , subthemes and illustrative quotes

| Themes and subthemes | Illustrative quotes |
| --- | --- |
| **1.Participants expectations and motivation for involvement** |  |
| **Prior experiences of involvement** | *I have never been engaged…except participation in interviews. I had experience of participation only related to this study…to Addis Ababa two times where I shared my experienced on large meeting at big hotel*…*When coming to this group I was expecting that they have planned to expand that more (previous capacity building training)…I expected that they might have planned to organize service user more so as to achieve our goal… (P2)*  *This was not my expectation at the onset…The facilitator gave us orientation and asked as what we would expect from the research…I remember only the training we had with Dr (…named) at Tabor hotel. Just like that, my expectation was one day short training; I did not expect that such action research where we go all through problem identification to solution (P5).*  *I expect to gain more from the study… because I was involved in the previous studies that lead to this research group. Starting from development of the roadmap that Dr (…named) facilitated…When I heard about this study, I cannot tell you how I was felt happy…(P3)*  *…at the beginning I was not that much motivated and expected something good. I appeared only to sit and see what would happen ... I had some concerns in the previous training, which was too long (whole day), very intense and was a bit boring. …[But] as sessions progressed, I realized that the PAR is actually doing about my own problems, we discussed and worked together something important for people with mental illness...Finally I said to myself “I should actively participate”. (P1)*  *I was expecting them (researchers) to ask me questions as they usual do. But this very different from the previous, we developed roadmap, established user organization and developed action plans. I am very happy. Totally different, I get what I did not expect; very interesting thing (P6)* |
| **Desire to learn and contribute something valuable** | *…That is education! To get educated, and learn from each other…I know there is education…that helps me to effectively care for my patient. I have no more than this expectation (P10)*  *… I have been struggling alone to solve many problems for long. There are many service-users who dropped out of healthcare service, and they were usually found around traditional and faith-based healers (Tsebel-Holy water). So, I have been thinking how to solve these problems, what to do, and whom to talk to… (P3)*  *…I felt painful when observing many service-users suffering just like I used to. I felt a sort of like the same bad experience. I have been in the same situation for 10 years. Now thanks to God I have passed that bad stage. So I want to contribute. I want to make things better for those suffering with mental illness (P2).* |
| 2. **Experiences of the social dynamics in the PAR process** |  |
| **Experience of activeness and inclusiveness of the PAR process** | *…At the initial sessions it seems there is difference. The more we enter to the discussion in the second, third sessions there is no visible difference in level of participation and ideas generated by health professionals, caregivers and service users…Our discussion was using the slogan “nothing about service-users without service-user”, there was not that much difference; because all of us had experience of the problem as family members, neighbors and as professionals… all of them were participating freely; even there were participants who entertain us presenting jokes… (P5)*  *…In my group (caregivers), the participation of all individuals was not the same. But we used to let everyone to express idea to his/her level of understanding. Everybody contributed to his/her level of understanding. There was no situation where anyone was passive observer and other individual dominate the discussion...everyone contributed his/her opinion. That was how we used to run the group discussion… (P1)*  *…Although we discussed in small group the idea generated was the same…That was very interesting and attractive. We share ideas where one has difficulty in understanding, others assist to clarify and explain ideas…The process was full of consensus…I have achieved my expectation and I am very happy… (P2)*  *…The participation was very interesting…there was small group session; all the group discussion was about idea construction. All groups generated good ideas and all sessions were summarized finally to generate action plan that was acceptable to participants (P4)*  *… There were individuals who actively participated as well as some silent individuals. But the facilitator (…named) encouraged everybody to speak more in a very entertaining ways and then all participated to the best of their level (P2).*  *The facilitator…‘operated’ very well to bring all together and discussed in a way that made all open to discuss idea. So my feelings were generally very positive about it. I used to anticipate the next session eagerly* (P3)  *I appreciated… used to encourage high level of involvement of all participants in the PAR process… accommodated everyone to have chance to express opinion. I think everybody got a time to reflect opinions. That is one of the reasons I committed and stay engaged in the PAR process (P5).*  *…That was very nice and did not waste my time. We utilized our time efficiently; because we come on time, engage in the discussion effectively and complete on time…because we scheduled it in public holidays (out of our working time)... We used to meet two days per week for two to three hours, which was very easy contribution of time (P1).*  *…it has been nice because they gave (facilitators) us some money. I appreciate being compensated for my time that definitely contributes to a more relaxed participation…It was because of this training and the financial compensation that I supported the patient…(P2)* |
| 3. **Perceived outcomes/benefits of the PAR process** |  |
| **Personal benefits** | *I gained knowledge that I lacked before. Now I have gained knowledge about different types of mental illness. Professor (…named) told us about existence of more than 300 types of mental illness. Before this, I used to understand mental illness as a single problem…I gained this knowledge. I gained knowledge about causes, about the treatment; I gained a lot of knowledge… (P1).*  *…The research is helping us… helped us to teach others… Before I come here, even, I don’t know my own problem, even I don’t know myself; I was living by chance. Now after the involvement in the research I can advise other people… (P6)*  *There was a lot of education in the group…we discussed ideas, freely exchanged ideas in small groups. We exchanged education from the facilitators and our presentations…The education works for service users...The education for caregivers was also prepared us to give necessary support to patients… We can identify people chained at home through education….I got additional knowledge on what is in me before.….Now I can care for my patient, my children and my neighbors. I don’t be careless about myself. Education has many benefits. Now I can teach my neighbors during coffee ceremony and at work place. I advised them to go to healthcare service and take medicine and medicine can help for recovery. Now I am teaching how to safely use medicine (P10).*  *I personally gained more direction that improves my professional work from what the service users and caregivers discussed. The caregivers discussed important issues. Now I don’t focus on their illness. I have learned about the importance of Compassionate Respectful Care (CRC) service; I feel health professionals have to effectively practice CRC. I have realized the importance of giving adequate information about medication use and the side effects for service-users. I have learned from the service-users the importance of delivering them necessary information about the service…increased my sensitivity to many points that I used to ignore or give less attention. For example, I did not pay attention whether there is comorbid case or not? Other issues I should ask about the needs of the patient (P3).*  *That was very interesting. First, I improved my writing skill. Second, I improved my self-confidence. Third, I have improved my ability to express my views to others. (P8)*  *The PAR process taught me a lot. First, I noticed that I can generate useful ideas from beginning to the end of all sessions; there were several thought provoking ideas discussed during the research process that creates ‘Ha!’ here am correct and I have created sense of being of value. Second, I have gained knowledge about how to develop action plan; how to start planning, with whom to work, about sources of support…HA! … This is not only for the study, but it is very important for personal life. I have learned how to live planned in my personal life. (P3)*  *The participation process had education and advice in it, which by itself can be considered as getting the medicine…For mental health I am taking my medicine properly. I have developed the confidence to take medication without fear of side effects. I am taking advice from health professionals about all side effects…I have great improvement after getting this experience in many aspects of my life. I have improvement. I am happy. I have hope. Thanks to God now I am health. I have recovered in health… thanks to God, I am living in hope. I am relaxed... Thanks to God…I have passed that difficult/crisis time, that dark time; now I am in light… (P2).*  *He (her husband) used to move outside and roam on street during nights and I have to move with him to protect him from danger. Now after involvement he has developed hope and I approach him and advise him at home. He has improvement and developing hope very much. We are surprised that human beings can bring change if given hope. The man was a person who attempted to hang himself a number of times and attacked others. This is why we see hope. The program is very helpful. I get good knowledge…Even I recovered from the pain I used to feel on my leg and hand (P7)*  *Now I am leading my life effectively. Previously I used to worry, I was much stressed. Involvement in the research has helped me a lot. I am leading my life and caring about my children. I am keeping my home very well. Previously I feel stressed. There was time I wished to die. (P6)*  *Personally I am calm and don’t like talking much and I had difficulty to speak in front of people; now after getting involved in the research group I have improvement and started talking at home and social gatherings (P6)*  *…That helped her very much. She had no training before and lacked knowledge; after the training and involvement in the research she gets improved much more. For example, before she did not speak more in public, now she started expressing herself very well in group discussion; … I saw her asking and speaking in social association(Idir)…,during coffee ceremony at home and neighborhoods she started teaching about the causes and problems of mental illness. At home she clearly describes about the topics that we covered during the training. These were not observed before, so this was one of practical change I observed… (P1)*  *…We become united… Because of the participation in the research group we have got freedom, now we are organized as service user association…We started saving money in bank…We started saving that can strengthen our relationship. I am very happy. (P2)*  *…Now we are organized, started saving and they (Sodo district officials) are facilitating the process of the registering the service user association… (P10)*  *We established service user association...I liked that and I am happy about the organization. Because we can meet and discuss together…and give home-to-home peer service and study about mental illness. (P6)*  *…One of the hopeful gains is the established service user association. More than what we put in our stomach (eat) and pocket better to support this hopeful organization. We are expecting the licensing of the service user association. (P7)* |
| **Social benefits** | *…I consider participation in this group as my rebirth. This is the chance I missed in my entire life. Now thanks to God, I am about 40 years old…I get the chance to sit with health professional;… I have been a person discriminated and neglected for life time…Now other people started appreciating the improvements observed on myself; they are saying to me “you are really getting young”. They say “she become new person… improved” talking at my back. When I hear this, I feel deep satisfaction... I keep my hygiene, dress very well and enjoy with my children. I dress my hair well and just I am free. Thanks to God…This is new beginning of my life… (P2)*  *…There is change, we get chance to know each other, know new people, there were different discussion events, this is new, we get all the weekends new things. We share for those we did not get the chance to involve… I don’t know I have positive feeling being there; meeting people and discussion with others makes me very happy. (P9)*  *…I had no chance to go out of home had it been for this research. Now I have developed many friends, passed my time with many people. This is creating association. Had it been for this research, I would have been locked at home with children. Involvement in the research benefited me a lot. Yes that is true it helps for developing social relation, now have many friends…(P6)*  *…With whom I discuss my idea…; no other people to discuss whether hope or grievance…no one to talk to. Thanks the illness of my husband opened this chance to learn this education. In my effort to support my husband I get the chance to learn. I get the opportunity to meet other people and communicate with them. Sharing idea and discuss ones problem with other people have solution... an individual who have no this opportunity is live-dead person. Discussion with other people is treatment by itself, it can bring idea and solution to problems and it can bring change. Individual left alone at home means that individual remains ignorant… (P7)*  *…This helped me to improve my relation and communication with people; the value people give has improved. This makes me happy… I had the chance to discuss with people from Addis Ababa University. I developed skills of participation with people, sitting with others and working with others. I used to pass locked at home; passing time here is very interesting. This is leisure time and recreational…there was no such opportunity…this helped me not to remain hidden or neglected. Here I have freedom to share my ideas. I have nice times here… Because of my participation here, my family is happy. (P8)*  *…Yes, changed for good. When I go to kebele, municipality,…and all other related areas, people are very happy about my improvement. Because previously no one trust me to hold money for a day (use it for alcohol). Now I am feeling responsible… when my friends see me, they ask me are you the one who changed like this. We enjoy together, chat together… We are not the same to yesterday. Enough! That is. We passed that enough! (P4)*  *…I have received feedback from different people. I have presented the finding of the research to representatives of different community and government offices. Our findings touch every sector, gender office, health office and psychiatry and others. People appreciated my presentation and commented that I was unnecessarily quiet for long. There were good things. (P3)* |
| **Benefit to research and the health care system** | *We are teaching others to get the benefit we get. They have to learn from the program with hope just like we did. That is the benefit I see…There are many important occasions to create awareness raising activities, for example during coffee ceremony with our neighbors, “Senbetie- Sunday gatherings”, Mahiber(traditional religious monthly festival) as well as Idir (traditional social association) for both males and females. These are important occasions we could use to teach many people. (P7)*  *…In addition to the lesson I get here, I shared a lot from my lived experience to change and motivate people to take their medication properly. In my neighbors I have supported many people to take medication properly and told them that they could recover through proper use of medicine. Even if the patients resist taking the medicine, caregivers need to help them take through negotiation. For many people, I personally, gave education. I have tried all my best. (P10)*  *...We have clearly discussed with health professionals about their problem in patient care, receptiveness, medication availability and …we discussed many issues. After I get involved in the research I had visit to the hospital for another individual and for my child, and I noticed that they are doing well…There is some improvement that can be appreciated. (P6)*  *…Yes there is change. As to my close follow up, in the hospital there is a psychiatric professional. He is really very committed to help. There are times he is attached by patients. But he encourages them and gives professional support. The other health professionals including the physicians and the health officers also started collaborating… (P4)*  *…Before involvement in the research, health professionals had self-distancing or pushing behavior towards mental health service users. We (health professionals) used to say the psychiatric nurse`s (…named) people came (mental service users)… consider only the psychiatric nurse in charge of the mental health service. More recently we are working with him at the psychiatric unit. This collaborative work needs to be strengthened. We had discussion to implement the findings of the research and what we have agreed in public with the hospital management bodies. They reassured as their willingness and there is good beginning now. (P5)*  *… I did not implement at hospital level because the study was complete more recently and we did not get much time to implement in the hospital…There are issues to be improved around health professionals. For example, the healthcare to mental health service users needs to start from the hospital gate; the current service has gaps in this regards. They should be treated like people with other health conditions, the attitudinal problem of health professionals need to be addressed in our hospital; I have taken responsibility to work on this issue. For example, we have planned to discuss with case teams and change teams to discuss about this. (P3).*  *…recently we have presented the findings of the research to large stakeholder groups that included both district government sector offices and community representatives. We had discussion with them how to sustain the research process and activities. If we are able to work sustainably with these stakeholders including religious institutions, schools teachers and students we can bring change. The community stakeholders who participated in the workshop can support and build capacity. The hospital management body including the medical director and CEO participated in the final stakeholder consultative meeting and discussion on research participant group finding dissemination. They have agreed to integrate the research participant group in the health education mainstream routine of the hospital. (P5)*  *…There is some change; now roadmap has been developed with stakeholders. A range of stakeholders that included religious leaders, education sectors, community associations (Idir), health professionals and all others were get some training. Now, there is good beginning. (P1)*  *...First we have learned working with stakeholders including health professionals, health office, and other community stakeholders. For example, last time soon after the end of the research group sessions we presented the findings to community stakeholders. We discussed on the findings with community stakeholders. This gave us opportunity to familiarize ourselves to enter and work with different community stakeholders as mental health unit this was great success. This was one great opportunity we familiarized ourselves to the community stakeholders. For example, as health problems I have several problems to identify and work with community stakeholders in practice. Previously we had problems of collaborative working with stakeholders; medication problem was another challenge… (P3)* |
| **Implementation challenges and recommendations** | *Our immediate problem is office where we meet in. For the time being we agreed to meet in open space in Buie hospital compound. The hospital administration has planned to give us office...If we are going to be localized in the hospital, I don’t think we get more attention. Hope the town municipal administration will collaborate with this…The other issue is about budget; now we don’t have budget as we are newly founded. Budget problem can deter us. Any association needs support to be well founded and sustain itself. We need money for transportation and other logistical issues including daily allowance for our members contribute to the community. Members need at least refreshment. These are potential problems that need immediate solutions. But I don’t expect these should be solved in short, and we have planned to solve gradually in our last meeting. In general I wanted to raise these as problems…these are problems. (P1)*  *…For the time being I don’t see more challenge to implement our plan. However, there are logistical challenges including budgets to compensate for time for people who are going to attend meeting. This can be a challenge and this was also potential challenge discussed in the research process. (P5)*  *I repeatedly visited the relevant administration office in the district to facilitate the registration of the association. But it is still challenging and took a lot of time for them to respond to and endorse the registration of the service user association. There was little practical support and I am completely giving up hope. I find that they are not moving as they promised during the workshop. (P4)*  *There is a tendency to push the healthcare service for people with mental health conditions to the psychiatric nurse alone. There are trained professionals in mhGAP, but are not allocated to the psychiatric service unit…the system has been working as it was for years… (P5).*  *The current study was a bit narrow. First, it seems had limited budget to involve more people over longer time. This is one important area that needs improvement. Second. The political leader should make mental illness a mainstream routine activity. Unless mental illness issue was supported with political leaders and government, this research group alone with external assistance… may not last long …and be able to bring sustainable change…Government has to put hand in this initiative… Therefore, as ownership to these initiatives our local government needs to give attention, allocate budget, take it as mainstream agenda, otherwise I don’t think this would be sustainable…the involvement of political leaders in this initiative need to improve...(P1)*  *I ask for consistent supply of medicine. There must be adequate supply of medicine at hospital and health centers, this is my basic question. We have asked this question previously, because there are people who cannot afford to pay. Now I am at good health status, I can dress well, make my hair and keep my personality. But there are people, who are in chain; who are totally out of conscious. Therefore, there must be adequate supply of medication… (P2)*  *... During the road map development and PAR process, I have realized that the mental health touches and needs working with many people including community organizations (Idir, Iqub). We have planned to work collaboratively with different organization and get their support…However…we need support for more sustainable work. Now we have the direction and the knowledge because of our actual involvement in the research. There is acute shortage of medicine in the hospital. The psychiatric nurse (…named) has been traveling to Worabe, Butajira and Addis Ababa to purchase drugs. Because of shortage of medication many people are on relapse and back to problem situations… There is discussion in the community advisory board to strengthen service user association. We have started saving money in the bank account. But sustainable support mechanisms are needed to strength the association to able to support its members as do the associations for other health conditions. (P5)* |
|  |  |
|  |  |
